# A New Concept to Reveal Protein Dynamics Based on Energy Dissipation

**DOI:** 10.1371/journal.pone.0026453

**Published:** 2011-10-17

**Authors:** Cheng-Wei Ma, Zhi-Long Xiu, An-Ping Zeng

**Affiliations:** 1 Institute of Bioprocess and Biosystems Engineering, Hamburg University of Technology, Hamburg, Germany; 2 School of Life Science and Biotechnology, Dalian University of Technology, Dalian, China; University of Akron, United States of America

## Abstract

Protein dynamics is essential for its function, especially for intramolecular signal transduction. In this work we propose a new concept, energy dissipation model, to systematically reveal protein dynamics upon effector binding and energy perturbation. The concept is applied to better understand the intramolecular signal transduction during allostery of enzymes. The *E. coli* allosteric enzyme, aspartokinase III, is used as a model system and special molecular dynamics simulations are designed and carried out. Computational results indicate that the number of residues affected by external energy perturbation (i.e. caused by a ligand binding) during the energy dissipation process shows a sigmoid pattern. Using the two-state Boltzmann equation, we define two parameters, the half response time and the dissipation rate constant, which can be used to well characterize the energy dissipation process. For the allostery of aspartokinase III, the residue response time indicates that besides the ACT2 signal transduction pathway, there is another pathway between the regulatory site and the catalytic site, which is suggested to be the β15-αK loop of ACT1. We further introduce the term “protein dynamical modules” based on the residue response time. Different from the protein structural modules which merely provide information about the structural stability of proteins, protein dynamical modules could reveal protein characteristics from the perspective of dynamics. Finally, the energy dissipation model is applied to investigate *E. coli* aspartokinase III mutations to better understand the desensitization of product feedback inhibition via allostery. In conclusion, the new concept proposed in this paper gives a novel holistic view of protein dynamics, a key question in biology with high impacts for both biotechnology and biomedicine.

## Introduction

As proteins are central to cellular function, researchers have sought to uncover the secrets of how these complex macromolecules execute such a fascinating variety of functions. Although static structures are known for many proteins, the functions of proteins are governed ultimately by their dynamical characters. Proteins are inherently dynamical molecules that undergo structural fluctuations over a wide range of timescales, from femtoseconds to milliseconds or longer [1], [2]. Structural fluctuations that occur on the fastest (femtosecond to picosecond) timescales permit the protein to sample a rugged energy landscape and ultimately facilitate slower, larger scale protein rearrangements that are responsible for modulating its biological function [3], [4], [5].

As a typical dynamical model for investigating the relationship between protein structure and function, allosteric proteins have attracted researchers' attentions for decades (for reviews see [6], [7], [8], [9]). Variant dynamical models have been proposed to discover the mechanism underlying the allosteric regulation. Among them, the population shift model has been widely accepted as a successful model to understand allosteric regulation. However, there is still one important question that needs to be addressed: how the signal is transferred upon the binding of a ligand to a regulatory site. Unfortunately, the answer to this question could not be obtained from previous models. New models are required to give a better interpretation of allosteric regulation and to get a deeper insight into the process of intramolecular signal transduction. In this paper, we propose a novel model which is called energy dissipation model. The aim of this work is to discover the energy dissipation model based on the allosteric process and to investigate this process by examining the characteristics of the energy dissipation model.

As respect to the technologies related to the investigation of protein dynamics, improvements have been made during the past few years. Besides experimental approaches, such as NMR relaxation [10], [11], [12], ultra-high resolution low-temperature X-ray crystallography [13] and Ultrafast laser technologies [14], molecular dynamics simulations of protein dynamics and allostery offer the opportunity to explore mechanistic details that are difficult to observe experimentally [15], [16]. A non-equilibrium technique called anisotropic thermal diffusion has been reported that monitors the effect of an artificially large but local temperature fluctuation as it diffuses through a protein structure [17]. Coarse-grained methods and normal mode analysis may be used to delineate the correlated conformational fluctuations of a protein structure in a model-independent manner [18], [19]. An ensemble-based approach has been applied to staphylococcal nuclease, and revealed a high degree of coupling between local structural fluctuations, ligand binding and global conformational changes [20]. In this work, specific molecular dynamics simulations are designed and carried out to simulate the input of external energy and to investigate the subsequent energy dissipation process using *E. coli* aspartokinase III as a model system.

**Figure 1:**
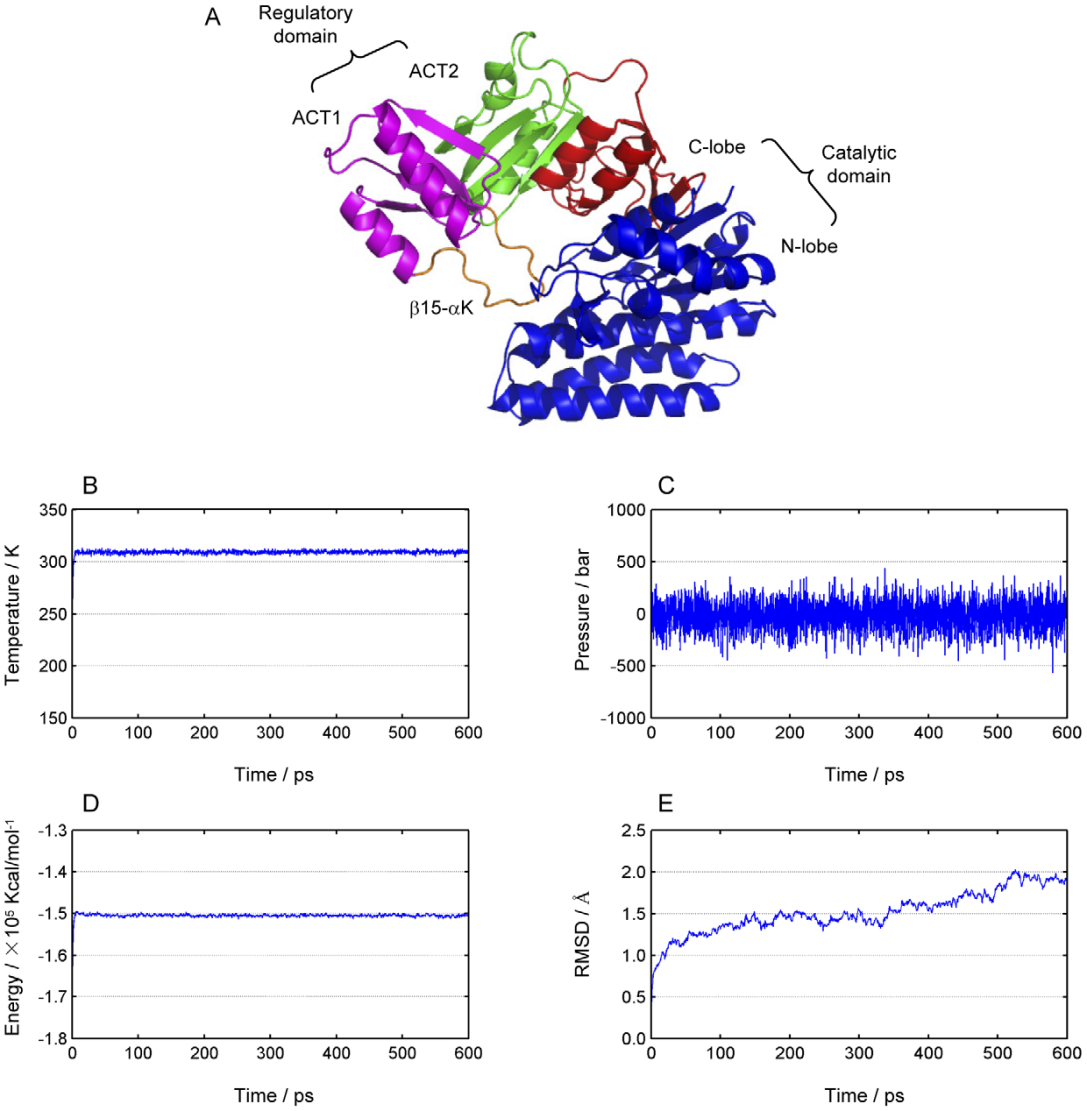
Structural regions of *E. coli* aspartokinase III and system parameters monitored during the molecular dynamics simulation. (A) The structure is organized into a C-terminal regulatory region and an N-terminal catalytic region. The C-terminal regulatory region consists of two ACT domains, in which the second ACT domain (colored in green) is inserted within the first (colored in purple) via connections in two β-strands. ACT1 exhibits the fold of a typical ACT domain with an extended 14-residue loop between β15 and αK (colored in orange). The catalytic region exhibits a typical amino acid kinase family fold which can be further divided into the N-terminal lobe (N-lobe, colored in blue) and the C-terminal lobe (C-lobe, colored in red). (B) Temperature of the system. (C) Pressure of the system. (D) Total energy of the system. (E) Backbone root-mean-square deviations (RMSD) calculated from the trajectory using the first configuration as the reference and all coordinate frames from the trajectories were first superimposed on the initial conformation.

Aspartokinase is a key regulatory enzyme which plays an important role in the synthesis of aspartate derived amino acids such as lysine and threonine. Small molecules like lysine or threonine are separately or jointly bound at the regulatory regions, causing a conformational change which in turn results in a change of the protein from an active state to an inactive state and the loss of enzyme activity. *E. coli* aspartokinase III (EC 2.7.2.4) is monofunctional and allosterically inhibited by lysine [21]. The subunit is organized into a C-terminal regulatory region and an N-terminal catalytic region (Fig. 1A). The C-terminal regulatory region consists of two ACT domains, in which the second ACT domain is inserted within the first via connections in two β-strands. ACT1 exhibits the fold of a typical ACT domain with an extended 14-residue loop between β15 and αK. ACT2 is made up of a C-terminal βαββα-fold and a β-strand N-terminal to ACT1, β12, completing the ACT domain architecture. The catalytic region exhibits a typical amino acid kinase family fold with an eight-stranded, mainly parallel β-sheet sandwiched by two layers of α-helices [22], which can be further divided into the N-terminal lobe (N-lobe) and the C-terminal lobe (C-lobe). The residue-residue interaction network involved in signal transduction in the allostery of *E. coli* aspartokinase III has been recently studied by a combined approach of evolutionary statistic analysis and molecular dynamic simulation [23]. Several mutations have been successfully developed to alter the allostery of aspartokinase III by the effector lysine. The application of the energy dissipation model proposed in this work to the *E. coli* aspartokinase III and its mutations revealed hereto unknown dynamic features and a possible second signal transduction pathway in this enzyme.

### Theoretical background and the new concept

Weber was the first to propose that the process of ligand binding merely shifts the population of the conformational states in the dynamic ensemble of the protein [24]. This has been substantiated by recent experiments showing that conformational states in the pre-existing equilibrium can influence protein function [25], [26], [27]. Population shift or re-distribution of protein conformational states is a powerful concept for rationalizing binding mechanisms and allosteric regulation [28], [29], [30], [31]. It has expanded the understanding and definition of allostery: (i) it emphasizes that rather than only in two conformational states, proteins exist in ensembles; (ii) it recognizes that allostery is a thermodynamic phenomenon; (iii) the existence of multiple conformational and dynamic states implies multiple pathways through which the strain energy is released from the allosteric site following a perturbation event.

In order to understand how the signal is transferred upon the binding of a ligand to a regulatory site, we propose a new model. This model is based on the following facts: (i) protein is an open system, energy can be transferred from external environments through intermolecular interactions (i.e. caused by a ligand binding); (ii) residues in proteins are fluctuating; (iii) the regulatory process is conducted by intramolecular non-linear interactions; (iv) the conformational distribution of a protein with or without ligand is in a quasi-equilibrium state while the signalling process is in a non-equilibrium state. According to our energy dissipation model, the allosteric process can be described as follows: (i) an allosteric protein is in an initial conformational distribution; (ii) external energy is introduced into the allosteric site when the ligand binds to it; (iii) the input energy dissipates through intramolecular interactions within the protein; (iv) the allosteric protein reaches a new conformational distribution. The new model regards the statements of population shift model as the prerequisite. However, it emphasizes that after the allesteric process of the open protein system is stimulated, the energy perturbation will pass through within the protein in a dissipative way, resulting in a re-distribution of protein conformational states.

To describe the characteristics of the energy dissipation process within proteins, the two-state Boltzmann equation (Eq. 1 and Eq. 2) is employed:
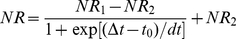
(1)

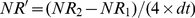
(2)


Where Δ*t* is the response time, *NR* is the number of residues affected by external energy perturbation during the energy dissipation process, *t*_0_ is the time point when half of the total number of residues have been affected, *dt* is a time constant, *NR*_1_ is the initial number of affected residue(s) and in this work it is set to be 1 unless statements, *NR*_2_ is the final number of affected residues and it is fixed to be 447 for *E coli* aspatokinase III, *NR*' is the first derivative at (*t*_0_, (*NR*_1_+*NR*_2_)/2), which reflects the change rate of the number of affected residues at this time point. Boltzmann equation was originally proposed to describe the possibility distribution of different states in an ensemble in statistical thermodynamics. The two-state Boltzmann equation is often employed to describe sigmoid curve behavior encountered in molecular biology [32]. It is applied here because of its ability to well describe the exponential stage of a sigmoid curve with more meaningful parameters than most of the other formal mathematical models.

For the change of the energy dissipation processes between aspartokinase III mutations and the wild-type, the Lorentz equation (Eq. 3 and Eq. 4) is used to describe the peak curve.
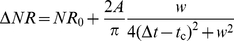
(3)

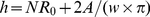
(4)


Where Δ*t* is the response time, Δ*NR* is the change of affected residue numbers during the energy dissipation process, *NR*_0_ is the offset, *t*_c_ is the time point when the largest change (*h*) appears, *w* is the width of the peak curve and *A* is the area under the curve. Because of the meaningful parameters that could be obtained from the Lorenz equation, it is employed here to describe the changes of the energy dissipation processes for different mutations in comparison with the wild-type.

## Materials and Methods

### Structures

The X-ray diffraction structures of *E. coli* aspartokinase III were retrieved from the Protein Data Bank (PDB, [33]). The crystal structure of the R-state (PDB code 2J0W) was employed to conduct the molecular dynamics simulations followed by energy dissipation simulations as well as to indentify the key amino acid residues that interact with substrates in the catalytic domain. The crystal structure of the T-state (PDB code 2J0X) was used to identify the key residues which have interactions with the effector in the regulatory domain. To get the structures of aspartokinase III mutations, residues of the mutation sites (V339A, R305A, S315A and Q308A) were mutated using the Mutator module embedded in the software of VMD [34], which is able to generate the residue structure that interacts with its neighbors in the best manner.

### Preparation

After deleting the substrates binding to the catalytic site, the crystal structure of the R-state was neutralized by adding sodium and chlorine ions with an ionic concentration of 0.5 mol L^−1^ and solvated in a rectangular box of TIP3P [35] water molecules with a minimum solute-wall distance of 10 Å. The solvated systems were energy-minimized by 5000 steps employing the software of NAMD [36] prior to the molecular dynamics simulations in order to relax the loops and side chains to make them suitable for performing the simulations.

### Molecular dynamics simulations

The aim of this step is to obtain the equilibrium state of the R-state protein, whose population is the highest in the initial ensemble. Molecular dynamics simulations were performed with a periodic boundary condition in the NPT ensemble using Langevin dynamics at 310 K with the damping coefficient of 5.0 ps^−1^ and constant pressure of 1 atm. The non-bond pair list was updated every 10 steps and the Particle Mesh Ewald (PME) method [37] was used to treat long-range electrostatic interactions. A residue-based cut-off of 12 Å was applied to the non-covalent interactions. No constraint was applied to the protein during the molecular dynamics simulations. A time step of 2 fs was used and the coordinates of the simulated complexes were saved every 1.0 ps. The simulations lasted 600 ps and were performed employing the software of NAMD with the CHARMM27 force field. Analysis of the molecular dynamics trajectory was conducted on the entire simulation to ensure the dynamical stability of the system. The backbone root-mean-square deviations (RMSD) were calculated from the trajectory using the first configuration as the reference and all coordinate frames from the trajectories were first superimposed on the initial conformation to remove any effect of overall translation and rotation. To examine the convergence of the molecular dynamics simulations, energy, temperature and pressure were monitored during simulations.

### Energy dissipation simulations

According to the energy dissipation model proposed in this work, if the energy of key residues of the regulatory site is changed, the energy perturbation will be transferred to the catalytic site through intramolecular non-linear interactions. This energy dissipation process will show a unique pattern which can reflect the dynamical characteristics of the protein. In this work, the energy of key residues residing in the regulatory site of aspartokinase III was changed by increasing its velocity.

To avoid involvement of unexpected force or energy, the energy dissipation simulations were conducted with a time step of 1 fs under the condition that the temperature and pressure of the system were able to change automatically during the simulation process. Other simulation parameters were the same as that used in the former step. The energy of each amino acid residue was captured during the entire dissipation process and the simulations lasted till all residues had responded to the energy perturbation. In the meanwhile, reference simulations in which the velocities of the residues were not changed were carried out to simulate the molecular dynamics process when no external energy was input to the protein. The energy change of each residue caused by the energy perturbation was then calculated by subtracting the energy of the reference simulations from that of the energy dissipation simulations.

## Results and Discussion

**Figure 2:**
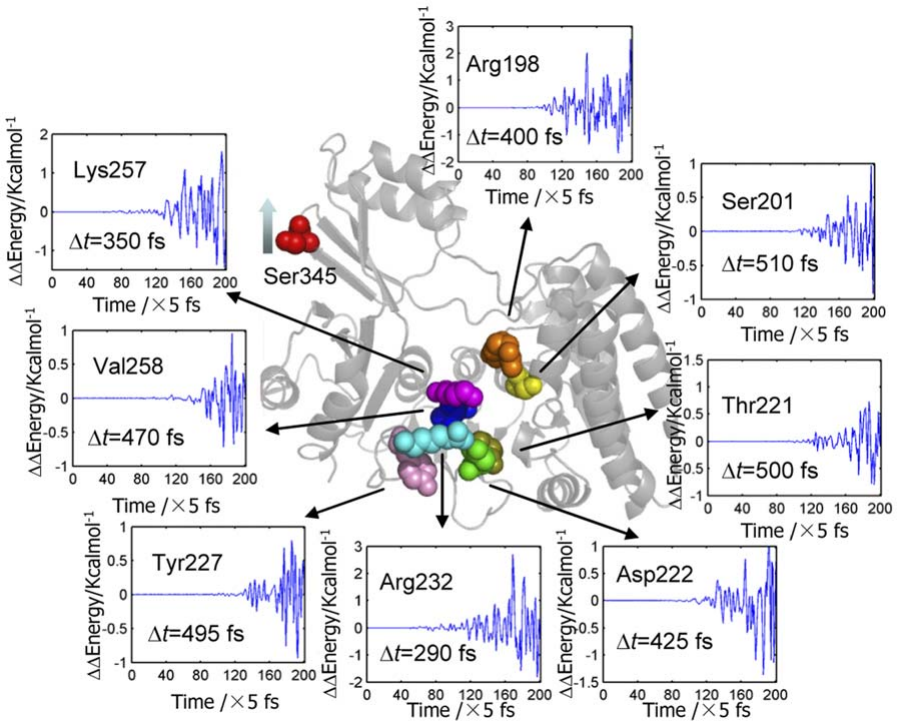
Energy changes upon energy perturbation. The velocity of Ser345 is increased by four fold in this case and only the energy changes the key residues belonging to the catalytic site are shown as examples. The key residues are shaped in sphere and differently colored; the other part of the enzyme is shaped in cartoon and colored in light gray. Δt is the time point when the residue responses to the energy perturbation.

According to the energy dissipation model, residues will response to the energy perturbation at different time points after the external energy input, forming the dynamic basis for signal transduction. To simulate the signal transduction process within *E. coli* aspartokinase III upon the binding of the effector lysine, conventional molecular dynamics simulations were first carried out to obtain the equilibrium state of the R-state protein (Fig. 1B–E). Then the velocity of Ser345, one of the key residues of the effector binding pocket, was increased by four fold. As examples, the energy changes of the key residues of the catalytic domain are shown in Fig. 2. As expected, the residues response to the energy perturbation at different time points, from 290 fs for Arg232 to 510 fs for Ser201. It is believed that the difference in their response time depends on the intramolecular non-linear interactions which are based on the component and structure of the protein and thus is employed here to reflect the protein dynamical characteristics.

### Energy dissipation curve

**Figure 3:**
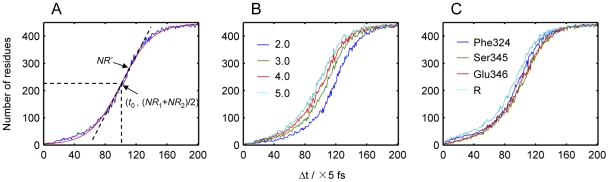
Energy dissipation curves of *E. coli* aspartokinase III. (A) Energy dissipation curve on condition that the velocity of Ser345 is increased by four fold (blue line) and the two-state Boltzmann equation fitting result of the energy dissipation curve (red line). *NR*' is the first derivation at the center point. (B) Energy dissipation curves on condition that the velocity of Ser345 is increased by different folds. (C) Energy dissipation curves on condition that the velocity of different residues is increased by four fold. R stands for the case when the velocities of all of the three key residues are increased by four fold at the same time.

The first characteristic of the energy dissipation process is the number of residues that has responded to the energy perturbation at different time points. This response can be defined as the energy dissipation curve for the whole protein. Fig. 3A shows the result when the velocity of Ser345 was increased by four fold. The energy dissipation curve exhibits a sigmoid pattern, which can be divided into three stages. During the first and third stage, the protein residues responded to the energy perturbation with a linear rate, while with an exponential rate in the second stage. The two-state Boltzmann equation described the energy dissipation curve satisfactorily (Fig. 3A). The parameter *x*_0_ in Eq. 1 is the time point when half of the residues have responded to the energy perturbation and thus is defined as the “half response time”. For *E. coli* aspartokinase III, half of the residues had been influenced at the time point of 501.4±1.1 fs. Therefore, its half response time is 501.4±1.1 fs. Another key parameter is the first derivative (*NR*') of the energy dissipation curve at the time point of half response time. It describes the exponential rate of the energy dissipation process and is thus defined here as the “dissipation rate constant”. The dissipation rate constant for *E. coli* aspartokinase III is 1.1 residues fs^−1^. This value means about another residue will respond to the energy perturbation at the time step of 1.0 fs. The half response time and dissipation rate constant are the most important parameters for the energy dissipation model to reflect the dynamical characteristics of the energy dissipation process in proteins.

### Energy dissipation at different energy perturbations

In order to investigate the influence of different strengths of energy perturbation upon the energy dissipation process, the velocity of Ser345 was increased by two, three, four and five fold, respectively. As can be seen in Fig. 3B, every energy dissipation process appears as a sigmoid pattern and the major difference is the second stage of the energy dissipation curves. The more energy was input into Ser345, the faster the energy was transferred to other residues as reflected by the parameters obtained from Boltzmann equation (Table 1). With the increase of the energy perturbation, the half response time decreases from 585.4±1.1 fs for the smallest energy input to 476.6±1.1 fs for the largest energy input. However, the dissipation rate constant remains the same (1.1∼1.2 residues fs^−1^) for all of them, indicating the fact that the dissipation rate constant describes a dynamical character determined by the component and structure of the protein rather than the strength of external energy input.

**Table 1:** Parameters of the two-state Boltzmann equation for characterization of the energy dissipation process when the velocity of Ser345 is increased by different folds.

Velocities (fold)	*t*_0_ (fs)	*dt* (fs)	*NR*' (residues fs^−1^)#	*adj-R* ^2^
2.0	585.4±1.1*	95.0±1.0	1.2	0.997
3.0	531.0±1.1	97.4±0.9	1.1	0.997
4.0	501.4±1.1	100.3±0.9	1.1	0.997
5.0	476.6±1.1	100.4±0.9	1.1	0.997

# : Calculated according to Eq. 2 using the mean values.

*: Standard error.

### Energy dissipation upon perturbation of different residues

Since there are several residues that bind the effector and then transfer the signal from the regulatory site to the catalytic site, it is necessary to examine the energy dissipation process when the energy is input to the other key residues and even to all of them at the same time. According to the crystal structure of the T-state of *E. coli* aspartokinase III, there are three residues (Phe324, Ser345 and Glu346) that interact with the effector. Thus, the energy dissipation processes for each of them were investigated on condition that their velocities increased by four fold and the results are given in Fig. 3C. Their energy dissipation curves appear to be very similar to each other, even for the case where the energies of all the three key residues were increased at the same time. The values of the half response time from the Boltzmann equation (Table 2) indicate that the signal was transferred the fastest (447.1±1.4 fs) when the energies of all the three key residues were increased at the same time. However, its dissipation rate constant (1.1 residues fs^−1^) is the same as that of the others.

**Table 2:** Parameters of the two-state Boltzmann equation for characterization of the energy dissipation process when the energy of different residues is increased.

Residues	*t*_0_ (fs)	*dt* (fs)	*NR*' (residues fs^−1^)#	*adj-R* ^2^
Phe324	474.7±1.3*	98.1±1.1	1.1	0.996
Ser345	501.4±1.1	100.3±0.9	1.1	0.997
Glu346	493.6±1.0	103.7±0.9	1.1	0.998
Phe324, Ser345, Glu346†	447.1±1.4	101.8±1.2	1.1	0.996

# : Calculated according to Eq. 2 using the mean values.

*: Standard error.

† : *A*_1_ is set to be 3 in this case.

### Residue response time

**Figure 4:**
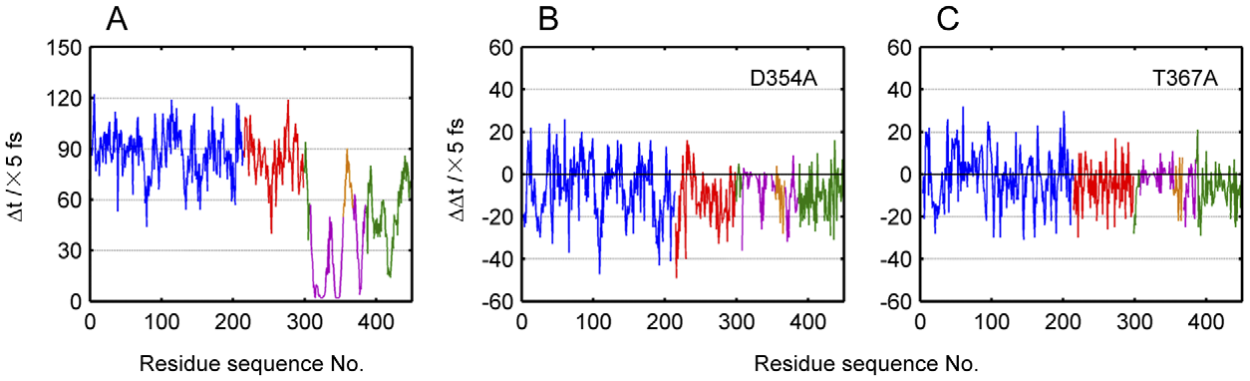
Residue response time of *E. coli* aspartokinase III. (A) Response time of each residue on condition that the velocity of Ser345 is increased by four fold in this case. Change of residue response time for mutations D354A (B) and T367A (C) compared with the wild-type under the same conditions. The curves are colored according to its structural regions: N-lobe in blue, C-lobe in red, ACT1 in purple, ACT2 in green, the β15-αK loop in orange.

The energy dissipation curve characterizes the energy dissipation process at the level of the whole protein. However, information at the level of residues is required in order to get a deeper understanding of the mechanism underlying the intramolecular signaling process. Thus, the response time of each amino acid residue was calculated and shown in Fig. 4A.

In Fig. 4A, the curve is colored according to different structural regions of *E. coli* aspartokinase III. It is evident that most residues of the regulatory region responded to the energy perturbation earlier than those in the catalytic domain. It also can be seen that residues belonging to different structural domains may respond to the energy perturbation at the same time point, indicating that the response time could provide novel information about the dynamical characteristics of the protein that could not be obtained from its structural information. For instance, residues of the N-lobe respond to the energy perturbation during the same time range as that of the C-lobe, although residues of the N-lobe are father away from the regulatory site than those of the C-lobe. This indicates that the signal may be transferred to the C-lobe and N-lobe through at least two different pathways at the same time. Besides the pathway provided by ACT2 (green colored line in Fig. 4A), there is another pathway existing in ACT1 between residues 354–367 (orange colored line in Fig. 4A).

To demonstrate the role of the residues 354–367 in the signal transduction process, Asp354 and Thr367 were mutated to alanine. The changes of their residue response time are shown in Fig. 4B and Fig. 4C. It could be seen that, for the two mutations, residues response to the energy perturbation earlier than the wild-type, indicating their important role in the signal transduction process. Table 3 lists the mean residue response time of the two mutations in detail. It could be found that the signal transduction process was influenced by changing not only the dynamical characteristics of the residues in C-lobe but also the residues belonging to ACT2. Further investigation of the second pathway will be given in the next section.

**Table 3:** Mean residue response time of different structural regions for the wild-type as well as mutations D354A and T367A.

	Mutation sites		
Structural regions	Wild-type	D354A	T367A
All	356.0±142.3#	313.9±142.4	337.8±143.0
Regulatory domain	206.3±128.3	163.9±120.2	169.1±111.7
ACT1	146.1±127.6	109.3±106.3	129.8±114.4
ACT2	270.6±93.6	223.2±105.7	231.8±81.0
Catalytic domain	432.3±70.3	390.2±92.5	418.8±71.3
C-lobe	420.0±71.6	358.8±71.3	391.0±73.5
N-lobe	437.2±69.3	402.6±91.5	429.8±67.8

(Unit: fs).

# : Standard deviation.

### Protein dynamical modules

We introduce the term “protein dynamical modules” in this work based on the residue response time upon energy perturbation. Residues responding in a certain time interval after the perturbation are classified into different dynamical modules. Different from the protein structural modules which merely provide information about the structural stability of proteins, protein dynamical modules could shed light on protein characteristics from the perspective of dynamics.

**Figure 5:**
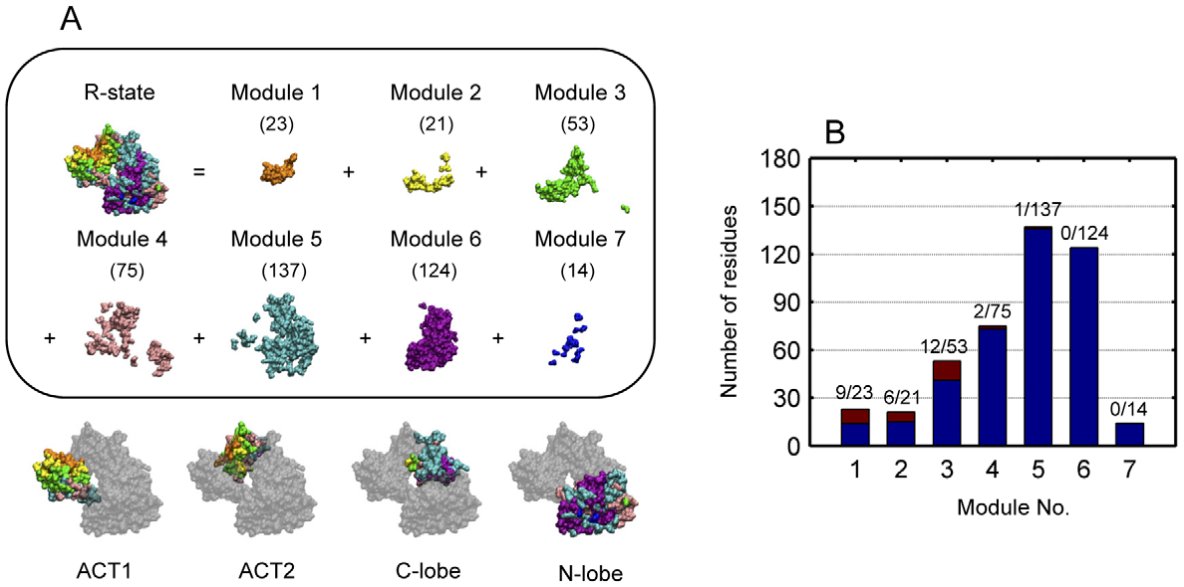
Protein dynamical modules of *E. coli* aspartokinase III and distribution of mutation sites. (A) Protein dynamical modules are obtained at a time interval of 0.1 ps. Modules are differently colored and numbers of residues within each dynamical module are given in brackets. (B) Numbers of residues within each protein dynamical module (colored in blue and brown, denominator) and distribution of mutation sites predicted by integrating molecular dynamics and co-evolutionary analysis [23] among each module (colored in brown, numerator).

As shown in Fig. 5A, the energy dissipation process under study can be divided into seven modules at a time interval of 0.1 ps. It shows how the signal is transferred within *E. coli* aspartokinase III. It also can be seen that residues belonging to the same protein dynamical modules may reside in different protein structural modules. The residues of the N-lobe responded to the energy perturbation during the same time range as those of the C-lobe, which is in agreement with the results given in last section. By combining the protein dynamical information with the protein structural information, we confirmed that the signal was transferred to the catalytic site with the following two pathways: one is the ACT2 pathway and the other is the ACT1 β15-αK loop pathway. These results provide the dynamical mechanism for the prediction proposed by Kotaka et al. [38] that binding of lysine to the regulatory ACT1 domain in R-state aspartokinase III instigates the release of a “latch”, the β15-αK loop, from the catalytic domain, which in turn undergoes large rotational rearrangements, promoting the transition to the T-state.

Among the protein dynamical modules, modules 5 and 6 contain more than half of the residues of *E. coli* aspartokinase III with module 5 containing the most. Compared with the energy dissipation curve in Fig. 3A, it is found that these two modules reside in the exponential stage. Thus, more residues will response to the energy perturbation at the same time interval. In addition, the key residues of the catalytic site, which interact with the substrates and thus determine the catalytic properties of *E. coli* aspartokinase III, belong to different protein dynamical modules although they reside in the same structural domain. This means that those key residues respond to the signal through different mechanisms, which is an important characteristic that could be used in the re-design of allosteric enzymes. As a preliminary illustration, this dynamical characteristic is employed to investigate *E. coli* aspartokinase III mutations in the following section.

### Application to different mutations

In a recent work from our group [23], a co-evolutive and predictive approach, which is based on sequence information of the protein family and structural information of a number of member proteins, was employed to engineer allosteric regulation of *E. coli* aspartokinases III. The allosteric properties were altered as desired to reduce the strength of a regulator. However, this approach depends on the quantity and quality of sequence information obtained for the protein family. Furthermore, it cannot provide dynamic information about mechanisms for the predictive results, which will limit the further development and application of this method. Thus, it is necessary to investigate the desensitization of allosteric inhibition employing the energy dissipation model.

In this work, the distribution of the mutation sites predicted by Chen et al. [23] among the dynamical modules was examined as given in Table 4 and Fig. 5B. Of the 30 mutations sites, 27 belong to the first three modules with module 3 containing the most; while none was found in module 6 and 7. This suggests the importance of the first three modules, especially module 3, in the signal transduction process of *E. coli* aspartokinases III and its potential application to the re-design of new allostery.

**Table 4:** Distribution of mutation sites predicted by integrating molecular dynamics and co-evolutionary analysis [23] among the protein dynamical modules.

No.	Mutation sites	Module No.	Reference	No.	Mutation sites	Module No.	Reference
1	M251P	4	[23]	16	E346R	1	[23]
2	T253R	3	[23]	17	V347M	1	[39]
3	R305A	2	[23]	18	V349M	1	[39]
4	S315A	1	[23]	19	T352I	2	[23]
5	M318I	3	[39]	20	T355	3	-
6	H320A	2	[39]	21	C378	3	-
7	G323D	3	[39], [40]	22	I392	5	-
8	F324	1	-#	23	F407	3	-
9	L325F	2	[39]	24	N414	4	-
10	F329R	3	[23]	25	R416A	1	[23]
11	I337P	3	[23]	26	M417I	1	[39]
12	S338L	3	[23]	27	S423	2	-
13	V339A	2	[23]	28	S424	3	-
14	T344M	1	[40]	29	N426	3	-
15	S345L	1	[39], [40]	30	C428R	3	[23]

# : Mutations of the corresponding positions have not yet been reported.

**Figure 6:**
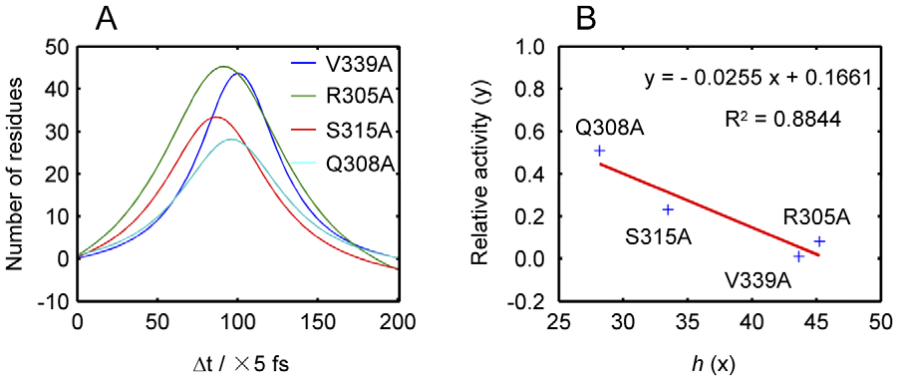
Influences of different *E. coli* aspartokinase III mutations and its linear correlation. (A) Characterization of the influences of different mutations on their energy dissipation curves using Lorentz equation. (B) Linear correlation between the relative inhibitions of aspartokinase III mutations and the *h* values derived from their Lorentz equations.

Secondly, mutations (V339A, R305A, S315A and Q308A) that showed different alternations of the intramolecular signal transduction and thus different levels of feedback inhibition (Table 5) were chosen for further investigation by simulating the energy dissipation process upon energy perturbation and then calculating the change of energy dissipation curves between the mutations and the wild-type. It is found that the largest changes appear during the second stage of the energy dissipation curve and the change curve within the whole timescale shows a pattern of peak curve. The Lorentz equation can well be used to characterize the peak curve as given in Fig. 6A and the parameters are listed in Table 5. It is evident that mutations with a higher degree of perturbation of the allosteric regulation resulted in higher *h* values. The *h* value is roughly linearly correlated to the relative inhibition of these mutations (Fig. 6B).

**Table 5:** Parameters of the Lorentz equation for the influences of different mutations on the energy dissipation process.

Mutation sites	*NR*_0_	*t*_c_ (fs)	*w* (fs)	*A* (fs)	*h* #	Relative inhibition*
V339A	−4.2±1.4†	502.0±4.5	311.5±24.0	23391.5±2045.0	43.7	0.01
R305A	−12.8±3.1	457.0±5.5	502.0±43.5	45763.5±5711.5	45.2	0.08
S315A	−7.1±2.2	433.5±7.0	411.5±44.0	26226.0±3600.5	33.5	0.23
Q308A	−4.0±2.0	481.0±8.0	401.0±50.0	20274.5±3235.0	28.2	0.51

# : Calculated according to Eq. 4 using the mean values.

*: Inhibition related to the wild-type of *E. coli* aspartokinase III.

† : Standard error.

The response time of the key residues of the catalytic site for both the wild-type and the mutations are listed in Table 6. It can be seen that the response time of Arg198, Ser201 and Arg232 to the energy perturbation was influenced by most mutations. However, different mutations influence enzymatic regulation through different mechanisms. For example, mutations of V339A and Q308A mostly changed the response time of Arg198 and Ser201 which belong to the N-lobe and are responsible for the binding of the aspartic acid, while R305A and S315A mostly influenced Thr221 and Arg232 which belongs to the C-lobe and is responsible for the binding of ATP.

**Table 6:** Residue response times of the key residues of the catalytic site for the wild-type as well as different mutations.

	Mutation sites				
Key residues	Wild-type	V339A	R305A	S315A	Q308A
Arg198	400	285	305	335	310
Ser201	510	415	420	445	370
Thr221	500	445	405	575	455
Asp222	425	460	470	505	485
Tyr227	495	420	490	400	410
Arg232	290	380	395	430	350
Lys257	350	385	390	400	385
Val258	470	480	410	450	455

(Unit: fs).

**Figure 7:**
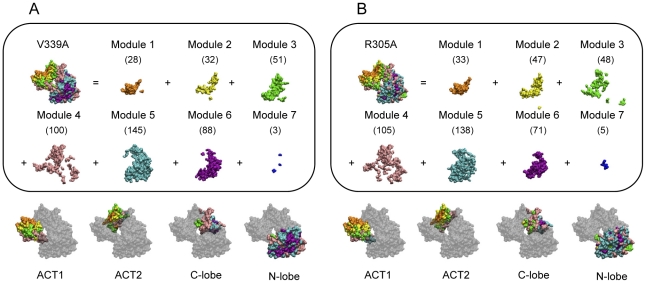
Protein dynamical modules of *E. coli* aspartokinase III mutations. Protein dynamical modules of mutations V339A (A) and R305A (B) are obtained at a time interval of 0.1 ps. Modules are differently colored and numbers of residues within each dynamical module are given in brackets.

At last, protein dynamical modules of *E. coli* aspartokinase III mutations V339A and R305A were calculated (Fig. 7) to compare with that of the wild-type. Comparing Fig. 7 with Fig. 5A, it is found that in all cases module 5 contains the largest number of residues and it changed slightly among them. The number of residues in modules 1, 2 and 4 increased; while the number of residues in modules 6 and 7 decreased. To investigate their difference in detail, the numbers of residues in each module for different structural regions are listed in Table 7. For module 1, the increase is caused by the increase in ACT1; for module 2, it is due to the increase in both ACT1 and ACT2; while for module 4, the increase is caused by the C-lobe and N-lobe. The decrease in the C-lobe and N-lobe results in the decrease in modules 6 and 7. For module 3, the number of residues in ACT1 decreases while it is increased in the C-lobe and N-lobe. The slight change in module 5 is the result of the decrease in ACT1 and C-lobe as well as the increase in N-lobe. Thus, it can be concluded that the changes in modules 1 and 2 are mainly determined by the regulatory domain; while changes in catalytic domain results in the differences in modules 6 and 7.

**Table 7:** Distribution of the residue numbers among different structural regions for each protein dynamical module of wild-type as well as mutations V339A and R305A.

		Module No.						
Mutation sites	Structural regions	1	2	3	4	5	6	7
Wild-type	ACT1	17	13	23	13	12	0	0
	ACT2	6	7	25	27	7	1	0
	C-lobe	0	1	4	13	40	24	2
	N-lobe	0	0	1	22	78	99	12
V339A	ACT1	21	18	18	21	0	0	0
	ACT2	7	14	26	19	7	0	0
	C-lobe	0	0	4	32	29	18	1
	N-lobe	0	0	3	28	109	70	2
R305A	ACT1	25	22	14	13	4	0	0
	ACT2	8	23	21	20	1	0	0
	C-lobe	0	1	8	36	25	14	0
	N-lobe	0	1	5	36	108	57	5

### General discussion about the new concept

Allosteric proteins undergo structural fluctuations over a wide range of timescales (from femtoseconds to milliseconds or longer). According to the ensemble theory, structural fluctuations over the wide range of timescales can be represented as different conformational states in an ensemble. According to the conformational redistribution model, there are active states, inactive states and all possible intermediate states in the initial ensemble. This feature makes the study of protein dynamical process difficult, because it is impossible to obtain all of the possible conformational states in the ensemble employing conventional molecular dynamics simulations. In fact, it is not necessary to investigate the dynamical process based on the whole ensemble. According to the population shift model, the population of the active states is higher than that of the others in the initial ensemble. Therefore, the active states could be chosen to demonstrate the dynamical process. Another advantage of this approach is that protein dynamical processes of different allosteric proteins could be compared if they are obtained according to the same strategy.

**Figure 8:**
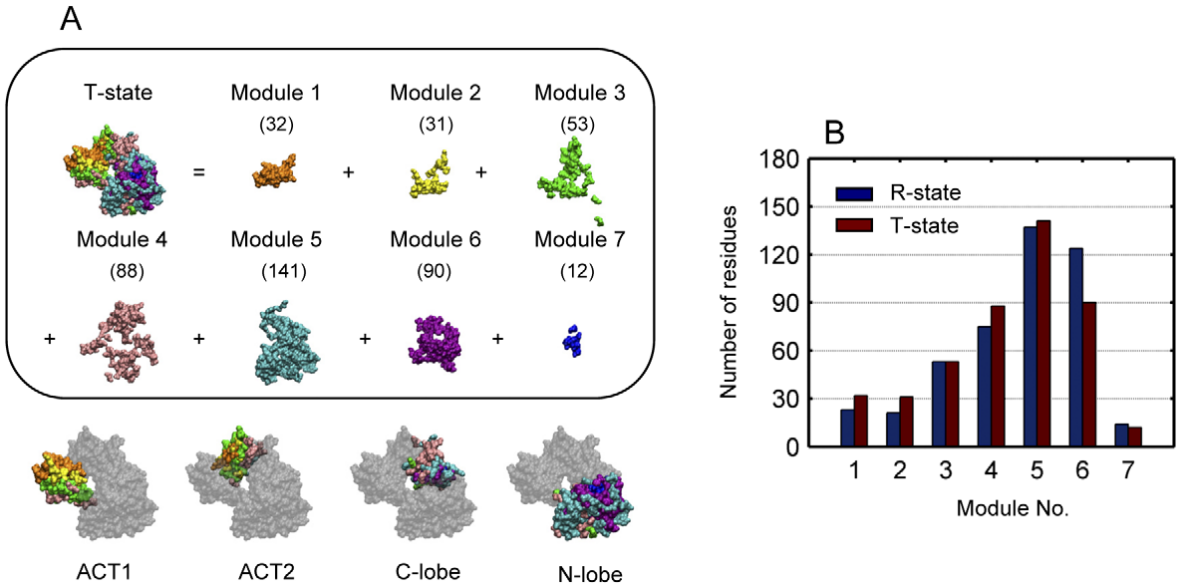
Protein dynamical modules of *E. coli* aspartokinase III using the T-state as the initial structure. (A) Protein dynamical modules are obtained at a time interval of 0.1 ps. Modules are differently colored and numbers of residues within each dynamical module are given in brackets. (B) Numbers of residues within each protein dynamical module obtained based on the R- and T-state of the protein.

Since protein dynamical modules proposed in this work are generated from the dynamical process, an extreme example, which used the T-state of the allosteric protein as the initial structure, is presented here to show how the dynamical modules are sensitive to different initial conformational states. Using the same simulation procedures and modularization approach, aspartokinase III was divided into seven dynamical modules, the same number as that based on the R-state of the protein (Fig. 8A). However, the number of residues is larger in modules 1, 2, 4 and 5, whereas it is smaller in modules 6 and 7 (Fig. 8B). Comparing the distribution of residues among the structural regions for each module (Table 5 and 8), it can be seen that the increase of the number in module 1 is due to ACT1. The increase in module 2 is caused by the whole regulatory domain (ACT1 and ACT2). The increase in modules 4 and 5 results from C-lobe and N-lobe separately. Meanwhile, the catalytic domain (C-lobe and N-lobe) results in the decrease of the number in module 6. These observations indicate that although the total number of modules may be the same, the number of residuals in each module and the distribution among the structural regions are usually quite different. On the one hand, useful information can be obtained from these differences (such as in the comparison of different mutations); on the other hand, same conformational states should be based on to generate protein dynamical modules (for instance, the active states) when different allosteric proteins are compared.

**Table 8:** Distribution of the residue numbers among different structural regions for each protein dynamical module obtained based on the T-state.

	Module No.						
Structural regions	1	2	3	4	5	6	7
ACT1	26	20	19	13	13	0	0
ACT2	6	11	26	22	8	0	0
C-lobe	0	0	4	25	36	18	1
N-lobe	0	0	4	28	97	72	11

In this work, protein dynamical modules were obtained by dividing the energy dissipation process at a time interval of 0.1 ps. Residues that responded to the energy perturbation at the same period of time were clustered into the same group (protein dynamical module). However, although this clustering method is straightforward, different clustering methods can be applied to the protein based on the data of residue response time to reveal the protein dynamics properties from different perspectives. The method used in this article only provides a simple way to cluster the data of residue response time. It would be interesting and meaningful to develop an automatic method or at least give a standard definition about how to obtain protein dynamical modules from the data of residue response time in order to make this concept more applicable.

How to simulate the effects of the ligand binding to proteins is a challenging task in the study of binding-induced biological processes. Although conventional molecular dynamics simulation does not encounter such problems, it is impossible to simulate the dynamics of intra-molecular signal transduction due to the large timescale of the process. Steered or targeted molecular dynamics simulations will introduce unexpected force or energy to the system, so they are not good choices either. Considering the fact that energy is the only transferable form during the binding process, finally we decided to simulate the binding process by increasing the energy of the key residues interacting with the ligand. However, the energy transferred to the binding pocket is conducted by forces such as von der Waals force and electrostatic force in the form of potential energy. Considering the facts that potential energy and kinetic energy transfer to each other during the process of protein dynamics (think of the quasi-harmonic approximation used in the study of protein dynamics) and it is easy to change the kinetic energy of atoms in molecular dynamics simulations, at last kinetic energy is chosen to simulate the binding process. Nevertheless, further strategies should be developed to simulate the binding process in the future.

### Conclusions

As it is expected, residues response to the energy perturbation at different time points, giving the energy dissipation curves in a sigmoid pattern. The half response time and the dissipation rate constant derived from the two-state Boltzmann equation can be used to well characterize the energy dissipation process. The more energy is input, the faster the energy is transferred to other residues. However, the dissipation rate constant remains the same, indicating the fact that the dissipation rate constant describes a dynamical character determined by the component and structure of the protein rather than the strength of external energy input. For the allostery of aspartokinase III, the residue response time indicates that besides the ACT2 signal transduction pathway, the β15-αK loop of ACT1 is another pathway between the regulatory site and the catalytic site. The distribution of the mutation sites among protein dynamical modules suggests the importance of the first three modules, especially module 3, in the signal transduction process of aspartokinases III and its potential application to the re-design of new allostery.
